# Œdema of the Glottis

**Published:** 1905-02-04

**Authors:** 


### ŒDEMA OF THE GLOTTIS.

A death from œdema of the glottis supervening on some septic condition of the neighbouring parts is an occurrence which is none the less unpleasant because unexpected, and, as a rule, the tragic termination is not anticipated either by the practitioner in attendance or by the friends and relatives of the patient. As pointed out by Sir Felix Semon,[1] although the progress towards disaster is usually rapid, there is a resemblance among different cases that is sufficient to make a definite clinical picture which, if once appreciated, will perhaps enable the practitioner in a particular instance to recognise the danger threatening his patient in time to make preparation for the tracheotomy which may be urgently required. Whether the septic process commences as a tonsillitis, a glossitis, a pharyngitis, or as the so-called angina Ludovici, or in any other way, the symptoms which precede œdema of the glottis are commonly much the same. Early in the case there is dysphagia, and this is soon succeeded by complete inability to swallow. At this stage inspection will show simple congestion, infiltration, or even considerable œdema of the parts. In many cases a particularly bluish, sometimes almost dark-blue, colour will warn the observer that the case is not an ordinary sore throat, and that something serious may be developing. The patient now soon becomes hoarse, this hoarseness in turn rapidly passing into complete aphonia. A few hours after the development of aphonia, dyspnœa commences, and quickly increases, necessitating tracheotomy, often enough within 24 hours from the onset of the first symptoms. Unfortunately, in the really severe cases the affection does not cease at the larynx, so that tracheotomy is often of no avail to save the patient. It is very rare for infection to extend up into the naso-pharynx, nose, or meninges, the usual course being for the inflammation to travel downwards, causing pneumonia. When the larynx is the site of the initial lesion, the pharynx usually escapes altogether. The infection has a tendency to attack the serous membranes of the body, the pleuræ, the pericardium, and—more rarely—the peritoneum. A characteristic feature of these cases is that while development takes place so rapidly, retrogressive changes ensue with equally astonishing rapidity. The larynx which is obstructed by swelling on the first day may show merely a wrinkled condition of the mucous membrane on the second. For this reason, and also because the disease may become arrested at any period of its course as suddenly as it began, although every case must be regarded as extremely serious, it need never be looked upon as necessarily fatal. The chief hope of more successful treatment in the future lies with serum therapeutics; though at the present time only a small percentage of cases is benefited by antistreptococcus serum.

[1] Brooklyn Med. Jour., Jan. 1905.

